# United in Pediatric Sports Medicine: Knowledge Between East and West Strategies Makes Us Better… Together

**DOI:** 10.1002/ars2.70173

**Published:** 2026-11-15

**Authors:** Henry Ellis, Ted Ganley, James Hui

In the spring of 2018, I was honored—and lucky—to pass Dr. Ted Ganley (current president of Pediatric Research in Sports Medicine [PRISM]) and Dr. James Hui (keynote speaker for PRISM) in the hallway for a brief introduction. Little did I know at that time that this would be my first introduction to the cultural importance of learning from one another—across borders and oceans—to better ourselves as surgeons and improve the treatment of our young athletes. At that year's PRISM meeting, Dr. Hui delivered an impressive keynote on his work on cartilage treatment in the knee, blending both basic and clinical science—a true pioneer in translational research. This experience made me realize the importance of uniting our ideas, treatments, research, strategies, and philosophies across hemispheres.

The concept of blending cultures, often described as East Meets West or West Meets East, carries the idea that one culture influences another—similar to a cooking show where Chinese‐American chef Ming Tsai teaches Asian‐European fusion cuisine on the Food Network, or when the musical sounds of American violinist Yehudi Menuhin merge with Indian sitar virtuoso Ravi Shankar. In medicine, this fusion—known as integrative medicine—continues to evolve because the Western approach (North American and European) focuses on disease‐based treatments, from medications to surgery, whereas Eastern traditions emphasize treating the mind and body as a whole system. Sports medicine, arthroscopy, and orthopaedics have greatly benefitted from this cultural blending.

From Japan's Professor Kenji Takagi's use of a cystoscope for knee arthroscopy in 1918 and his development of the first practical arthroscope in 1931 to Boston's Professor Lyle Micheli's innovations in treating prepubescent anterior cruciate ligament injuries,^1^ we have spent the past century advancing each other's work. Modern arthroscopy owes much to Professor Masaki Watanabe of the University of Tokyo, who pursued significant improvements in the procedure. Watanabe facilitated global progress by hosting many Western surgeons, including Robert W. Jackson, M.D. (Canada), and Richard O’Connor, M.D. (United States). The young specialty of pediatric sports medicine is just now developing its fusion between East and West.

Similarly, Professor James Hui has been a key figure in bridging Eastern and Western cultures. As Malcolm Gladwell once described, Dr. Hui from the National University Hospital/National University of Singapore is a connector—one of the best in pediatric sports medicine. His international work on translational medicine includes using mesenchymal stem cells for cartilage repair and anterior cruciate ligament treatments in adolescents, as well as clinical outcomes related to patellar instability, discoid meniscus, and osteochondritis dissecans. He has also actively participated in US‐led research on osteochondritis dissecans of the knee and frequently reaches out over social media, iMessage, or WhatsApp to discuss various topics such as association with malalignment in discoid meniscus or biologic augmentation for osteochondritis dissecans.

The relation between Hui and Ganley has been a catalyst for blending Eastern and Western perspectives in pediatric sports medicine. After a trip I took with Ganley to visit Hui in Singapore, he reflected that we shared a common thread—a bond forged by a commitment to evidence‐based medicine and an unyielding dedication to advancing the field for the benefit of children.^2^ Our shared vision has led to significant progress in injury prevention and innovative treatments, grounded in a mutual respect for our cultural, ethnic, resource, and philosophical differences. Together, we are uniting as a global medical community focused on the youth athlete and have much more work to do to fuse our knowledge gaps.

One area of interest that has fused a connection between East and West is a common pastime obsession shared by both cultures—baseball. Global efforts in injury prevention stem from the growing popularity of youth baseball. Challenging medical conditions, such as medial epicondyle apophysitis or avulsion or capitellar osteochondritis dissecans, have influenced both of our efforts to avoid injuries that may be career‐ending or have long‐term consequences. As early as 2006, Olsen (United States) identified key factors like pitch count, velocity, and mechanics associated with injury, leading to youth pitching recommendations.^3^ A decade later, research from Japan showed the efficacy of injury prevention programs for medial elbow injuries in young athletes using a randomized controlled trial.^4^, ^5^ Along the same lines, Takahara's work highlighted not only treatment strategies for capitellar osteochondritis dissecans but also the value of ultrasound screening to detect silent injuries in baseball players.^6^, ^7^ This early work has spurred both Western and Eastern interest in identifying osteochondral lesions before symptoms develop, fostering a cross‐cultural exchange of knowledge on preventing injuries in youth throwers.

Nearly 8 years after Hui's keynote at PRISM, he returned to the 2025 PRISM Annual Meeting in Columbus, Ohio, where he brought together international researchers including Dr. Andy Zhou Zhilin (China), Dr. Yuichi Hoshino (Japan), Dr. Chih‐Kai Hong (Taiwan), and Professor Qiwei Li (China) to present their work on common pediatric sports medicine conditions. During this meeting, and at the “East Meets West” instructional course, Hui and Ganley once again approached me with a plan to further fuse our research efforts and collaboration in pediatric sports medicine across cultures.

Today, our collaboration focuses on understanding the variations in characteristics and treatment strategies for conditions such as pediatric anterior cruciate ligament injuries, discoid meniscus, osteochondritis dissecans, patellar instability, and tibial eminence. Our experience working together solidifies our common thread in research and outcomes, but knowledge of our differences will unify us.

## DISCLOSURES

The authors (H.E., T.G.) declare the following financial interests/personal relationships which may be considered as potential competing interests: H.E. reports grants or contracts from the Pediatric Orthopaedic Society of North America, the American Orthopaedic Society for Sports Medicine, and the Department of Defense Washington University in St. Louis (payments made to the institution); payment or honoraria for lectures, presentations, speakers bureaus, manuscript writing, or educational events from Orthopediatrics (payments made personally); leadership or fiduciary roles with Pediatric Research in Sports Medicine (Vice President), Research in Osteochondritis Dissecans of the Knee (President), and the Texas Orthopedic Association (Past President, current board member); and financial or nonfinancial interests with Arthrex (nonfinancial educational support), DJO (food and beverage), Pylant Medical (educational fees), and Stryker (food and beverage). T.G. reports participation on the Advisory Board of the International Pediatric Orthopaedic Symposium and leadership or fiduciary roles with PLUTO Pediatric ACL National Study Group (founding member), Pediatric Research in Sports Medicine (cofounder), and Research in Osteochondritis Dissecans of the Knee (cofounder). The other author (J.H.) declares that he has no known competing financial interests or personal relationships that could have appeared to influence the work reported in this article.
